# Can Heparin-Coated ECMO Cannulas Induce Thrombocytopenia in COVID-19 Patients?

**DOI:** 10.1155/2021/6624682

**Published:** 2021-06-04

**Authors:** Barbara Steinlechner, Gabriele Kargl, Christine Schlömmer, Caroline Holaubek, Georg Scheriau, Sabine Eichinger, Johannes Gratz, Bernhard Rössler

**Affiliations:** ^1^Division of Cardiothoracic and Vascular Anesthesia, Department of Anesthesia, Intensive Care Medicine and Pain Medicine, Medical University Vienna, Vienna, Austria; ^2^Division of Hematology and Hemostaseology, Department of Medicine I, Medical University Vienna, Vienna, Austria; ^3^Division of General Anesthesia and Intensive Care Medicine, Department of Anesthesia, Intensive Care Medicine and Pain Medicine, Medical University Vienna, Vienna, Austria

## Abstract

Extracorporeal membrane oxygenation (ECMO) is often used in the management of COVID-19-related severe respiratory failure. We report the first case of a patient with COVID-19-related ARDS on ECMO support who developed symptoms of heparin-induced thrombocytopenia (HIT) in the absence of heparin therapy. A low platelet count of 61 G/L was accompanied by the presence of circulating HIT antibodies 12 days after ECMO initiation. Replacement of the ECMO system including cannulas resulted in the normalization of the platelet count. However, the clinical situation did not improve, and the patient died 9 days later. Careful consideration of anticoagulant therapy and ECMO circuit, as well as routine HIT antibody testing, may prevent a fatal course in ECMO-supported COVID-19 patients.

## 1. Introduction

About 5% of coronavirus disease 2019 (COVID-19) patients become critically ill and develop acute respiratory distress syndrome (ARDS) [1].

Extracorporeal membrane oxygenation (ECMO) is often used to manage refractory hypoxemia in severe cases of respiratory failure [2, 3]. Vienna General Hospital is an expert, high-volume ECMO center with widely available ECMO devices, trained staff, and vast experience in the field of severe cardiorespiratory failure and lung transplant.

Current Extracorporeal Life Support Organization (ELSO) guidelines recommend the continuous infusion of unfractionated heparin up to a rate of 20.0 units/kg/h as an anticoagulant therapy during ECMO [4]. However, heparin may trigger heparin-induced thrombocytopenia (HIT), a potentially fatal condition characterized by a decline in platelet count and, puzzlingly, an increase in thromboembolic events [5]. HIT is the result of a severe immune response mediated by the formation of IgG antibodies against heparin/platelet factor 4 (PF4) complexes. These immune complexes activate platelets and lead to platelet aggregation, thereby causing thrombocytopenia. In addition, the release of PF4 by activated platelets induces a massive production of thrombin, promoting a prothrombotic state [6, 7]. The incidence of HIT in severely ill patients may be higher than previously appreciated. In a study involving 300 ECMO-supported patients after cardiac surgery, Opfermann and colleagues found an HIT incidence of 7.3% with a 59% mortality rate [8]. A recent systematic review of the literature revealed an HIT frequency of 17% in patients on venoarterial or venovenous ECMO support [9]. In COVID-19 patients treated with intravenous unfractionated heparin for at least five days (without ECMO support), the incidence of the positive HIT immunoassay was 12% with a 60% mortality rate [10]. Surprisingly, there are few data available on HIT in ECMO-supported COVID-19 patients.

## 2. Case Report

We report the case of a 69-year-old woman admitted to a primary hospital in Vienna, Austria, presenting with fever, dry cough, headache, and diarrhea. The patient tested positive for COVID-19 by reverse transcription-polymerase chain reaction of a nasopharyngeal swab specimen. Eight days after admission, the patient's respiratory state deteriorated rapidly resulting in severe hypoxemia.

**Figure 1:**
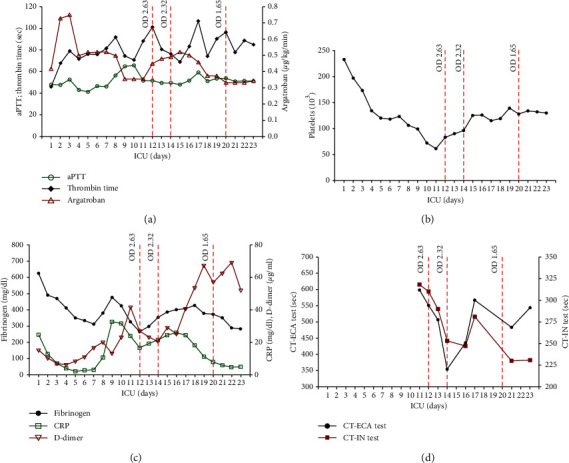
Clinical course in the ECMO-supported COVID-19 patient. (a) Anticoagulant argatroban was administered throughout the provision of venovenous ECMO. Anticoagulation effect. (b) Time course of platelets and results of the ELISA test (ZYMUTEST^®^ HIA IgG). Anti-heparin/PF4 antibodies detected by optical density (OD) read on days 12, 14, and 20 were monitored by measuring activated partial thromboplastin time (aPTT) and thrombin time (TT). (c) Time course of fibrinogen, D-dimer, and C-reactive protein (CRP). (d) Undulating course of coagulation time (CT) in intrinsic (IN) and ecarin (ECA) tests in contrast to the relatively uniform course of aPTT measurement.

She was unresponsive to noninvasive continuous positive airway pressure (CPAP) ventilation and nasal high-flow oxygen and was transferred to the intensive care unit (ICU), intubated, and prone positioned. Because of an unaltered low oxygenation index indicating severe respiratory failure, she was transferred to Vienna General Hospital for venovenous ECMO initiation (Cardiohelp System, Maquet Cardiopulmonary GmbH; Rastatt, Germany). A heparin-coated, double venous cannula system (BIOLINE coating, Maquet Cardiopulmonary GmbH; Rastatt, Germany) was used, with one cannula inserted into the right femoral vein and the other into the right internal jugular vein. Chest computed tomography scans showed multifocal bilateral patchy shadows indicative of COVID-19-related ARDS. Upon admission, the patient was on alternative anticoagulation with the direct thrombin inhibitor argatroban due to her allergy to low-molecular-weight heparins. Hence, the patient continued receiving argatroban at a rate between 0.33 and 0.73 *µ*g/kg/min as an anticoagulant therapy during ECMO provision. We relied on careful laboratory monitoring to guide argatroban dosage (Figure 1(a)). Lung-protective ventilation and therapy in the sense of “compassionate use” of IV immunoglobulins, anakinra (IL-1 inhibitor), and low-dose hydrocortisone were initiated.

On day 11 after ECMO initiation, swelling of the right leg was detected, caused by a nonocclusive thrombus within the right popliteal vein. Moreover, the patient presented with livid discolored fingertips. On day 12, the patient's platelet count had decreased to a nadir of 61 G/L (Figure 1(b)). Platelet count was checked with a specific blood collection tube (ThromboExact, Sarstedt, Nümbrecht, Germany) confirming the results collected with the citrated blood tubes and to rule out pseudothrombocytopenia (PTCP), an in vitro phenomenon of low platelet count caused by the agglutination of platelets, leading to false low platelet counts in automated cell counting [11]. Furthermore, antiphospholipid antibodies were excluded. The time course of the acute-phase proteins C-reactive protein and fibrinogen and elevated D-dimer levels indicating active blood clotting is shown in Figure 1(c).

The patient was highly positive for anti-PF4/heparin antibodies (optical density (OD): 2.63) as determined by the enzyme-linked immunosorbent assay (Figures 1(a)–1(d)), indicating a high likelihood of HIT. The widely used 4T's clinical scoring system was predicting a high probability of HIT (in sum, 8 points: 2 points in each category as (1) the degree of thrombocytopenia; (2) the timing of the platelet decline after heparin administration; (3) the presence of new thrombosis; and (4) nonapparent other causes of thrombocytopenia.

Since the heparin-coated ECMO cannulas were suspected to have triggered HIT, both the cannulas and the ECMO system were replaced by nonheparin-coated ECMO circuits (SOFTLINE circuits; Maquet Cardiopulmonary GmbH; Rastatt, Germany), resulting in the normalization of the platelet count within 4 days (Figure 1(b)). However, we could not revert the clinical situation. On day 22 after ICU admission, the ECMO indication was reevaluated, and the interdisciplinary team of the ICU and transplant surgeons decided on therapy de-escalation as the patient did not qualify for lung transplantation. Thus, due to nonrecovery and progressive organ dysfunction, the patient was weaned from ECMO support and passed away within a few hours

Lung autopsy results described diffuse alveolar damage (DAD) and pulmonary fibrosis in organization.

## 3. Discussion

Here, we report the first case of a patient with COVID-19 on venovenous ECMO support who developed symptoms of HIT in the absence of heparin therapy. The HIT diagnosis was based on clinical symptoms, including a low platelet count and thromboembolic complications, and supported by a highly positive anti-PF4/heparin antibody immunoassay test result (OD = 2.63). The patient was already on the alternative anticoagulant argatroban at the time of the ECMO implantation. Hence, we suspect that the heparin-coated ECMO cannulas may have triggered HIT in our patient with COVID-related ARDS. Consistently, the removal of heparin-coated cannulas led to a normalization of the platelet count within 4 days.

One complication of heparin therapy and heparin-coated circuits is HIT. Recently published data show that an OD threshold of 1.0 in anti-PF4/heparin antibody ELISA testing has a specificity of 89% and a negative predictive value of 95% for detecting/excluding HIT in ECMO patients on unfractionated heparin [12]. Therefore, our ELISA OD result of 2.63 together with the clinical features indicates a high likelihood of HIT. Until 2016, the functional heparin-induced platelet-activation (HIPA) test was performed at our institution in case of a positive ELISA test result. Based on receiver operating characteristic curves (ELISA versus HIPA test), we determined that the ZYMUTEST HIA IgG (HYPHEN BioMed) ELISA test had a sensitivity of 86% and specificity of 81% at OD levels >0.8. Therefore, an OD of 0.8 was established as a clinically relevant cutoff value for HIT, and HIPA testing was no longer performed [13]. Functional assays are still widely used as a confirmative test in the diagnosis of HIT. One of the most common tests is the platelet serotonin-release assay (SRA). However, recent data from an overall HIT cohort, without focusing on extracorporeal life support [14], and case reports indicate a certain unreliability of functional assays in ECMO patients [15].

Our patient was on alternative anticoagulation with argatroban, a reversible inhibitor of thrombin with a short half-life. A major concern with argatroban is subtherapeutic anticoagulation in patients with confounding elevated partial thromboplastin time in the presence of a nonspecific inhibitor (such as lupus anticoagulant) and secondary to additional coagulopathy in COVID-19 patients (increased levels of the VWF antigen, FVIII, D-dimers, and fibrinogen) [16]. For the first time at our institution, we have performed an ecarin (ECA) test at the later ICU stay of the patient (Figure 1(d)). Ecarin, a venom of the saw-scaled viper *Echis carinatus*, activates prothrombin in ClotPro® (Haemonetics GmbH, Munich, Austria), a new viscoelastic point-of-care testing device. We think that coagulation time in the ECA test could improve laboratory monitoring in patients on argatroban in addition to aPTT monitoring. This is being evaluated in an ongoing study at our institution.

Our data suggest that the heparin-coated circuit is a potential source of heparin exposure during ECMO provision. Covalently bonded heparin on the grafts and cannulas makes the circuits more biocompatible and limits the need for anticoagulation therapy during ECMO, particularly when there are bleeding concerns [17]. Although heparin-bonded devices may contribute to the development of HIT in previously unaffected patients, the medical guidelines are not very clear regarding the routine change of heparin-coated devices in case of suspected HIT [18, 19]. Centers of Excellence (ELSO) follow different strategies. In some facilities, ECMO cannula and circuit change is performed very early after the suspicion of HIT and the detection of anti-PF4/heparin antibodies, even if functional assays such as serotonin-release assays are negative [15]. Our department (cardiac, thoracic, and vascular anesthesia and intensive care medicine) changes the established ECMO circuit except cannulas in HIT-positive patients. This might be caused by the high percentage of VA-ECMOs at our ICU and thereby increased risk of adverse events during the exchange of cannulas. In this particular case, we assumed that the heparin-coated tube system triggered HIT since the patient was pretreated with argatroban and had no other documented exposure to heparin during her stay. This made a cannula and circuit change obligatory. Considering the published data, it is not comprehensible to which extent ECMO cannulas and circuits are changed in HIT-positive patients in general, and data considering COVID-19 HIT-positive ECMO patients are scarce. However, there are reports of regular and standardized exchanges of heparin-coated systems for nonheparin-coated systems in COVID-19 patients with a HIT diagnosis [20]. Given our presented case, current recommendations and future treatment strategies need to be discussed and overthought.

The medical history of the patient's allergy to low-molecular-weight heparin and the infection with SARS-CoV-2, which is per se the cause of a procoagulant state, put her at high risk for the development of anti-PF4/heparin antibodies. Importantly, early HIT diagnosis may allow a timely change in the anticoagulant therapy and circuit and improve the clinical outcome.

Alternatively, endogenous heparin may have also been released by mast cells present in tissues in close contact with the external environment, including skin and airways, in response to the infection.

Intermittent flushing of heparin to prevent occlusion in long-term central venous catheters, heparin in the pressurization system for arterial lines, and heparin in prothrombin complex concentrates [21, 22] were all ruled out as potential sources of heparin.

We also considered other reasons for thrombocytopenia in COVID-19 patients on ECMO support. Patients requiring ECMO may also develop thrombocytopenia due to contact with foreign surfaces, sepsis, bleeding, or medications. Furthermore, SARS-CoV-2 infection may affect the normal platelet biology in many ways, from reducing platelet production to increasing platelet breakdown [23]. In summary, the origin of thrombocytopenia may have been multifactorial, reflecting both heparin-dependent and -independent mechanisms. Nevertheless, replacing the ECMO system and cannulas normalized the platelet count, which led us to think of HIT as the most likely explanation in this particular case.

COVID-19 and HIT are both prothrombotic conditions that, when intertwined, can have devastating consequences if the HIT diagnosis is missed. If COVID-19 causes HIT, or if the procoagulant state during the infection is caused by other platelet-activating mechanisms, is currently the object of further research [24–26]. Several health organizations recommend the use of ECMO support in COVID-19-related refractory hypoxemic respiratory failure, yet the risk of complications such as HIT is not fully established. Early reports suggested that mortality could be as high as 94% in ECMO-supported COVID-19 patients, compared to 71% in conventional therapy patients [27]. In contrast, a recent study based on ELSO registry data estimated a 90-day mortality of 38% in ECMO-supported COVID-19 patients, supporting existing recommendations [3]. Unfortunately, neither study provides data on anticoagulation therapy or thrombocytopenia occurrence.

HIT monitoring could help to reconcile these apparently conflicting results and, most importantly, prevent fatalities in ECMO-supported COVID-19 patients.

The way of confirming HIT diagnosis remains controversial. In our case, functional confirmative assays for the diagnosis of HIT were not available.

## Data Availability

The data used to support this study are available in the Patient Data Management System of the Vienna General Hospital, University Hospital, and the site of the Medical University of Vienna.
